# Antibacterial efficacy of *Moringa oleifera* leaf extract against pyogenic bacteria isolated from a dromedary camel (*Camelus dromedarius*) abscess

**DOI:** 10.14202/vetworld.2019.802-808

**Published:** 2019-06-14

**Authors:** Ehab Ali Fouad, Azza S. M. Abu Elnaga, Mai M. Kandil

**Affiliations:** Department of Microbiology and Immunology, National Research Centre, Egypt

**Keywords:** antibacterial activity, camel abscess, *Moringa oleifera*, pyogenic bacteria

## Abstract

**Background:**

Abscess in camel is one of the most important bacterial infections. It causes anemia and emaciation, resulting in an economic loss due to carcass condemnation and a decrease in reproductive and production efficiency.

**Aim:**

This investigation aimed to isolate the bacteria from abscesses in camels and evaluate the antibacterial activity of *Moringa oleifera* extracts.

**Materials and Methods:**

Disk diffusion method and minimum inhibitory concentration were used for the evaluation of the antibacterial activity of *M. oleifera* extracts against isolated bacteria from camel abscesses.

**Results:**

The isolated bacteria were displayed relatively as follows: *Corynebacterium* *pseudotuberculosis* (30.4%), *Staphylococcus aureus* (25.8%), *Escherichia* *coli* (17.8%), *Corynebacterium* *ulcerans* (10.5%), *Klebsiella* *pneumoniae* (8.5%), *Pseudomonas aeruginosa* (8.5%), *Micrococcus* spp. (6.7%), *Proteus vulgaris* (5.2%), *Citrobacter* spp. (4.2%), and *Staphylococcus epidermidis* (1.7%). The drugs of choice for *Corynebacterium* isolates were ciprofloxacin and trimethoprim/sulfamethoxazole, whereas amikacin, ciprofloxacin, gentamicin, neomycin, novobiocin, streptomycin, and vancomycin were for *Staphylococcu*s isolates. Moreover, the ethanol extracts of *M. oleifera* showed higher antibacterial efficacy than the cold aqueous extracts.

**Conclusion:**

*M. oleifera* is considered one of the new infection-fighting strategies in controlling pyogenic bacteria responsible for camel abscesses.

## Introduction

In the past, researchers paid only a little attention to camel research. However, numerous bacterial, viral, and parasitic diseases have been recorded. Camel abscesses have received little attention with the bulk of research directed toward historic diseases of the camel. In addition, in abscesses, there are both economic and public health hazards, which generally result in economic losses due to the condemnation of infected carcasses or part of it, leading to nutritional problems [1]. Camels’ breeding could be done to overcome Egyptian’s needs for animal protein [2]. Certain breeds of camel can live in more humid environments; the major breed is the dromedary of the Nile Delta of Egypt [3].

There are few literatures on camel research, consequently, no recent data related to economic losses due to abscesses. However, abscesses in animals cause tremendous economic losses such as decreased production of animal meat, skin, and wool. Animals with abscess become anemic and emaciated, due to toxins produced from it, resulting in loss of the animal’s value and decrease in its reproductive and productive efficiency [4-6]. Infected animals had a poor physical condition, decreased fertility and condemnation of carcasses totally or partially at abattoirs [1,7,8].

Camel infections due to pyogenic bacteria such as *Corynebacterium pseudotuberculosis*, *Corynebacterium pyogenes*, *Streptococci* spp., *Staphylococci* spp., *Corynebacterium ulcerans*, *Rhodococcus*, *Escherichia coli*, *Citrobacter* spp., *Klebsiella pneumoniae*, *Proteus* spp., and *Pseudomonas aeruginosa* have been commonly reported in many areas [8-10]. In dromedaries, cervical and sciatic lymph nodes were mainly affected. Lymphangitis and suppurative lymphadenitis have been recorded in camel abscesses [11,12]. Camel abscess results in the entrance of the causative organism through the damaged skin and mucous membrane ended by reaching the regional lymph node then causes inflammatory and necrotic changes [13].

*Moringa* species is used in the medicinal field worldwide due to its pharmacological activities and considerable medicinal compounds. The most common species of *Moringa* genus is *Moringa oleifera* which has rich sources of various phytochemical compounds including glucosinolates and has antibacterial activity [14]. According to the World Health Organization, 80% of the populations in developing countries prefer to use herbal extract and their active components as traditional medicine therapy[4]. *M. oleifera* is commonly known as drumstick and has many active components such as alkaloids, tannins, flavonoids, saponins, and triterpenoids [15,16] with potent anthelmintic activity [17] and antibacterial effect [18]. The phytochemical analysis of *M. oleifera* showed its bioactive compounds [19,20] with their pharmacological activity [21]. *M. oleifera* has a broad safety margin for human and animal consumption [22]. Disk diffusion method was used for the evaluation of the antibacterial activity of *Moringa* extracts, and the significant difference of inhibition zones appeared. *P. aeruginosa*, *Enterococcus* *faecalis*, and *Staphylococcus aureus* were used to assess the antibacterial effect of the extract [18,23].

This investigation aimed to isolate the bacteria from abscesses in camels and evaluate the antibacterial activity of *M. oleifera* extracts.

## Materials and Methods

### Ethical approval

The study was performed according to the Guide for the care and use of Laboratory animals and Ethical Approval of Animal Rights according to Committee, National Research Centre, Egypt.

### Sampling

One hundred and seventy pus swabs were collected from 30 living and 70 slaughtered male camels (7-9 years old) that were obtained from El-Monib abattoir, Giza. These samples were obtained from external abscesses involving superficial lymph nodes and subcutaneous tissue, especially at the head, neck, and shoulder regions. Internal abscesses were mainly seen in the lungs, liver, and bronchial lymph nodes. According to Hatem *et al*. [5], the swabs placed in ice bags were transported through Cary–Blair (Difco) transport medium to the Department of Microbiology and Immunology, Veterinary Division, National Research Centre (NRC).

The collected swab samples were streaked onto blood agar plates (nutrient agar 13 g/L containing 5% citrated sheep blood), MacConkey agar plates (52 g/L), and mannitol salt agar 7.5% plates (111 g/L). All samples were streaked in duplicate plates and were incubated aerobically and anaerobically at 37°C for 24 h and 37°C for 48-72 h, respectively.

### Identification of bacterial isolates

Bacterial isolates were microscopically identified according to Cruickshank *et al*. [24]. Biochemical identification of Gram-positive coccobacilli was carried out according to Funk *et al*. [25]. Biochemical identification of Gram-positive cocci and Gram-negative bacteria was carried out according to Quinn *et al*. [26] and Cruickshank *et al.*, respectively [24].

### Antibiotic sensitivity test for identified strains

Fifteen standard antibiotic disks were used against the isolated bacteria after preparation of the standardized bacterial inoculums matching with 0.5 McFarland tubes (10^8^ colony-forming unit [CFU]/ml). Then, 25 µl of the inoculum was distributed on Muller–Hinton agar plates and incubated at 37°C for 24 h. The degree of sensitivity was determined by measuring the inhibition zone. The result was interpreted according to Dzotam *et al*. [16].

### Plant material of *M. oleifera*

*Moringa* leaf powder was obtained from Moringa Unit, NRC. It was collected from a farm of the NRC in Al-Nubaria which is situated along Cairo/Alexandria desert road. The plant material was collected in January 2018 and presented in the study.

### Cold aqueous extracts of *M. oleifera* leaves

100 g of *M. oleifera* leaf powder was weighed out and dissolved in 400 ml of cold distilled water into a conical flask stoppered with rubber corks and left for 7 days with occasional shaking (10 times/day). The mixture was filtered off using a sterile filter paper (Whatman no. 1) into a clean conical flask and subjected to water bath evaporation where the aqueous solvent was evaporated at its boiling temperature of 100°C. The standard extracts obtained were then stored in a refrigerator at 4°C for antibacterial activity test [27].

### Hot aqueous extracts of *M. oleifera* leaves

The same protocol as in cold water treatment was used with 30 min of boiling while the plant material was dipped in distilled water.

### Ethanol (95%) extracts of *M. oleifera* leaves

Here, the same procedure as in cold water treatment was followed.

### Test microorganisms

All the isolated bacteria from camel abscesses were used to assess the antibacterial effect of *M. oleifera*. It included *C. pseudotuberculosis, C. ulcerans*, and *S. aureus* as Gram-positive bacteria and *E. coli*, *K. pneumoniae, Citrobacter* spp., *Proteus vulgaris*, and *P. aeruginosa* as Gram-negative bacteria.

### Antibacterial assay of *M. oleifera*

The antibacterial activity of the three different samples, namely, (1) cold water extract (CWE) of leaves, (2) hot water extract of leaves, and (3) ethanol extracts (EEs) of leaves, was individually tested against the studied bacteria. *In vitro* antibacterial test was then carried out by disk diffusion method [28,29] using 25 µl of the standardized bacterial suspension of the tested bacteria (10^8^ CFU/ml) spread on plates. The disks (6 mm in diameter) were impregnated for different samples with 10 µl of 0.1 g/ml (100 mg/disk), followed by air drying, and were placed on seeded agar plates. Negative controls were prepared using the same solvents to dissolve the plant extracts. Tetracycline (TE) (30 µg/disk) was used as a positive control to determine the sensitivity of bacterial strain. The plates were incubated at 37°C for 24 h. The antimicrobial activity was evaluated by measuring the zones of inhibition against the tested bacteria.

### Minimum inhibitory concentration (MIC)

The MIC of different samples of *M. oleifera* was determined by two-fold serial dilution method [14]. Serial dilution of 100 mg/ml for rest of the samples were separately done to achieve 50, 25, 12.50, 6.25, 3.12, 1.56, 0.78 mg/ml and 390, 195, 97 µg/ml concentration were used for MIC determination. Briefly, 100 µl of varying concentrations of samples were added into the test tubes separately, containing 9 ml of the standardized suspension of tested bacteria (10^8^ CFU/ml). The test tubes were incubated at 37°C for 24 h. Controls were used with the test organisms, using distilled water instead of the plant extract. The least concentration of the samples with no visible growth was taken as the MIC [30].

### Statistical analysis

To evaluate associations between variables (antibiotic profiles), the data were analyzed statistically using Student’s “*t*-” test, showing mean + standard deviation [31].

## Results

The isolated bacteria were displayed relatively as follows: *C. pseudotuberculosis* (30.4%), *S. aureus* (25.8%), *E. coli* (17.8%), *C. ulcerans* (10.5%), *K. pneumoniae* (8.5%), *P. aeruginosa* (8.5%)*, Micrococcus* spp. (6.7%), *P. vulgaris* (5.2%), *Citrobacter* spp. (4.2%), and *Staphylococcus epidermidis* (1.7%). These bacteria obtained from 170 pus swabs were collected from 30 living and 70 slaughtered male camels (7-9 years old) that were obtained from El-Monib abattoir, Giza. *Corynebacterium* spp. colonies were appeared smooth, white in color, opaque, flat, circular, and small in size. Moreover, they had a narrow zone of ß-hemolysis on blood agar. The biochemical characteristics of *C. pseudotuberculosis* were positive catalase, urease, glucose, maltose fermentation and negative starch and trehalose fermentation, negative gelatin liquefaction, and nitrate reduction. *C. ulcerans* exhibited positive catalase, urease, gelatin liquefaction and fermented glucose, maltose, starch, and trehalose, with negative nitrate reduction test. On the other hand, Gram-positive cocci on nutrient agar was golden yellow in color; was smooth, opaque, circular, and medium in size; and was surrounded by the zone of ß-hemolysis on blood agar but was yellow in color on mannitol salt agar. While the non-hemolytic white colonies on blood agar exhibited pink colonies on mannitol salt agar. *S. aureus* was biochemically identified by catalase and coagulase positive, fermented maltose, trehalose, mannitol, and sucrose. While, *S. epidermidis* was coagulase negative, catalase positive and fermented sucrose only. *Micrococcus* isolates showed catalase positivity, coagulase negativity, and oxidative reaction (O) in the O/F test. Moreover, the Gram-negative bacilli were smooth, flat, circular and medium-sized colonies; either lactose fermenter colonies (appeared pink on MacConkey agar) or non-lactose fermenter (colorless). The biochemical characteristics of the isolated Gram-negative bacteria revealed the isolation of *E. coli* (17.8%), *P. aeruginosa* (8.5%), *K. pneumoniae* (8.5%), *P. vulgaris* (5.2%), and *Citrobacter* spp. (4.2%). The antibiotic sensitivity test is shown in Table-1.

**Table-1:** Antibiogram of reference drugs against bacterial isolates from camel abscesses.

Antibiotic	Bacteria					

	*Corynebacterium* (%)		*Staphylococcus* (%)		Gram-negative bacteria (%)	
		
	Sensitive	Resistant	Sensitive	Resistant	Sensitive	Resistant
Amikacin	85		100	-	95	-
Ampicillin	*	94	-	*	*	-
Augmentin	*	91	-	*	*	-
Ciprofloxacin	100	-	100	-	100	-
Gentamicin	86	-	100	-	-	90
Erythromycin	*	92	-	*	-	-
Metronidazole	-	87	-	70	-	100
Neomycin	80	-	100	-	*	
Novobiocin	85	-	100	-	80	-
Penicillin G		90	65	-	*	-
Rifampicin		79	86	-	-	82
Streptomycin	96	-	100	-	100	-
Trimethoprim/sulfamethoxazole	100	-	96	-	100	-
Vancomycin	-	100	100	-	-	100
Tetracycline	80	-	78	-	90	-

%=Was calculated according to the number of examined samples.

* Intermediate zone of inhibition

### Antibacterial activity of *M. oleifera* extracts

The antibacterial activity of cold water, hot water, and EE of *M. oleifera* is shown in Table-2. The EE of the leaves displayed a pronounceable better antibacterial effect against all the tested *C. pseudotuberculosis*, *C. ulcerans*, *S. aureus*, *E. coli*, *K. pneumoniae*, *Citrobacter* spp., *P. vulgaris*, and *P. aeruginosa* and their corresponding inhibition zone diameters were 25.65±0.04, 30.5±0.28, 26.75±0.04, 27.75±0.04, 28.5±0.3, 20.85±0.05, 24.75±0.12, and 22.25±0.04, respectively. The CWE of leaves showed relatively obvious antibacterial effect against *C. pseudotuberculosis*, *C. ulcerans*, *S. aureus*, *E. coli*, *K. pneumoniae*, *Citrobacter* spp., *P. vulgaris*, and *P. aeruginosa* with their individual diameter zones of inhibition recorded at 22.5±0.04, 25.5±0.12, 14.75±0.05, 18.25±0.28, 21.75±0.04, 20.65±0.13, 14.75±0.04, and 17.5±0.04, respectively. However, no inhibitory action was determined for hot water extract. The TE antibiotic was used as a positive control in comparison of *Moringa* extracts’ activity. The results showed the high antibacterial activity of ethanol and CWEs compared to the activity of TE.

**Table-2:** Antibacterial activity of *Moringa oleifera* extracts against some pyogenic bacteria.

Bacteria	Zone of inhibition (mm)			

	Cold water extract	Hot water extract	Ethanol extract	Positive control tetracycline
Gram-positive				
*Corynebacterium pseudotuberculosis*	22.5±0.04	+	25.65±0.04	18.3±0.12
*Corynebacterium ulcerans*	25.5±0.12	+	30.5±0.28	20.16±0.19
*Staphylococcus aureus*	14.75±0.05	+	26.75±0.04	17.20±0.13
Gram-negative				
*Escherichia coli*	18.25±0.28	+	27.75±0.04	20.16±0.43
*Klebsiella pneumoniae*	21.75±0.04	+	28.5±0.3	16.75±0.08
*Citrobacter* spp.	20.65±0.13	+	19.5±0.05	22.65±0.12
*Proteus vulgaris*	14.75±0.04	+	24.75±0.12	19.2±0.04
*Pseudomonas aeruginosa*	17.5±0.04	+	22.25±0.04	16.75±0.08

Values are presented as mean±S.E of triplicate experiments. +=Growth

Table-3 shows the MIC of the extracts from *M. oleifera*. MIC values of EEs ranged from 390 to 780 µg/ml, whereas the MIC values of the aqueous extracts ranged from 25 to 50 mg/ml. In this study, the lowest MIC value was exhibited by EEs at 390 µg/ml.

**Table-3:** MIC values of *Moringa oleifera* extract against some pyogenic bacteria.

Bacteria	MIC		

	Cold water extract mg/ml	Hot water extract	Ethanol extract mg/ml
Gram-positive			
*Corynebacterium pseudotuberculosis*	25	nd	390
*Corynebacterium ulcerans*	25	nd	390
*Staphylococcus aureus*	50	nd	390
Gram-negative			
*Escherichia coli*	25	nd	390
*Klebsiella pneumoniae*	50	nd	780
*Citrobacter* spp.	50	nd	390
*Proteus vulgaris*	25	nd	780
*Pseudomonas aeruginosa*	25	nd	780

nd=No detection, MIC=Minimum inhibitory concentration

## Discussion

*M. oleifera* is considered one of the new infection-fighting strategies in controlling pyogenic bacteria responsible for camel abscesses.

Improvement of camels’ health and breed should be kept in mind for the economic significance of camel meat and milk. [2]. The appearance of abscesses in camel creates a marketing problem due to the decline of the meat quality and quantity and condemnation of the affected portions and internal organs. Isolation of *Corynebacterium* species, *S. aureus*, *Streptococci*, *Rhodococcus*, *E. coli*, *K. pneumoniae*, *Citrobacter* spp., *P. vulgaris*, and *P. aeruginosa* from lymphadenitis and abscesses in camels had been reported in the literature [1, 8-12].

The present work focuses on the isolation and identification of bacteria causing abscess in camel. The results of the survey on the prevalence of abscesses in camels resulted in the isolation of *C. pseudotuberculosis* (30.4%), *S. aureus* (25.8%), *E. coli* (17.8%), *C. ulcerans* (10.5%), *P. aeruginosa* (8.5%), *K. pneumoniae* (8.5%), *Micrococcus* spp. (6.7%), *P. vulgaris* (5.2%), *Citrobacter* spp. (4.2%), and *S. epidermidis* (1.7%). *C. pseudotuberculosis* was the highest isolated one followed by *S. aureus* and *E. coli*. These organisms were previously reported as a cause of camel abscess by Wernery [6], Hassan *et al*. [7], Wernery [8], Berlin [11], and Wernery and Kinne [12]. The Gram-negative bacteria did not isolate alone in an abscess, but they were associated with *Corynebacterium* spp. and/or *Staphylococcus* spp. Further investigations are needed to know if they can induce abscess alone or they come as a secondary infection.

An antibiogram study was assessed on all isolates; Table-1 clearly indicates that ciprofloxacin (100%), trimethoprim/sulfamethoxazole (100%), streptomycin (96%), amikacin (85%), neomycin (80%), gentamicin (86%), TE (80%), and novobiocin (85%) were sensitive. Whereas vancomycin (100%), penicillin G (90%), metronidazole (87%), and rifampicin (79%) were resistant for the isolated *Corynebacterium*. This agrees with the finding of Hatem *et al*. [5], Hassan *et al*. [7], Muckle and Gyles [32], Judson and Songer [33], Zhao *et al*. [34], and Mohan *et al*. [35]. Table-1 indicates that vancomycin (100%), ciprofloxacin (100%), amikacin (100%), neomycin (100%), gentamicin (100%), streptomycin (100%), novobiocin (100%), trimethoprim/sulfamethoxazole (96%), rifampicin (86%), penicillin G (65%), and TE (78%) were sensitive to *Staphylococcus* isolates. This agrees with the finding of Hatem *et al*. [5], Lowy [36], Moran *et al*. [37], and Zhu *et al*. [38]. While Hatem *et al*. [5] recorded sensitivity to ampicillin (94.29%), augmentin (91.43%), and erythromycin (91.43%), this result disagrees with of the *Staphylococcus* isolates, as they were resistant to metronidazole, ampicillin, augmentin, and erythromycin. However, Guler *et al.*, Udo *et al.*, and Virdis *et al*. [39-41] illustrated the reason for lack of antibiotic susceptibility of *Staphylococcus* due to the multidrug-resistant genes acquired by bacteria. The multidrug-resistant strains of bacteria should be faced by new infection-fighting strategies [42,43].

**Figure-1:**
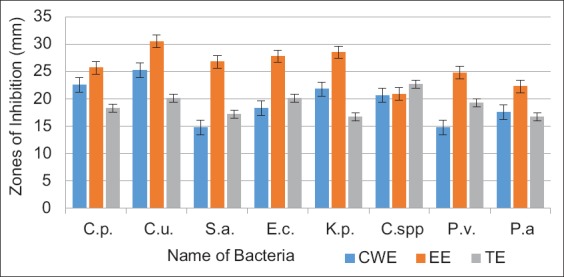
*Moringa oleifera* extracts and tetracycline antibiotic effect on some pyogenic bacteria. CWE=Cold water extract, EE=Ethanol extract, TE=Tetracycline, C.p.=*Corynebacterium pseudotuberculosis*, C.u.=*Corynebacterium ulcerans*, S.a=*Staphylococcus aureus*, E.c.=*Escherichia coli*, K.p.*=Klebsiella pneumoniae*, C.spp.=*Citrobacter* spp., P.v.=*Proteus vulgaris* and, P.a=*Pseudomonas aeruginosa*.

The present investigation illustrated that the disk diffusion method was used for the evaluation of the antibacterial activity of *M. oleifera* extracts. The EEs showed higher antibacterial effect than the cold aqueous extracts (Figure-1). Hence, the extraction process with an organic solvent such as ethanol provided a better antibacterial activity than the processes of soaking, obtaining decoction, and boiling of the plant in water, which agrees with the finding of Nair *et al*. [44]. In this investigation, the greatest inhibition zones were found in EE against all the bacteria tested, which were higher than that of antibiotic TE (30 µg/disk) (Figure-1). In earlier studies, researchers reported that plant extracts showed less antibacterial effect on bacteria [45,46]. The pharmacological activities and considerable medicinal compounds of *M. oleifera* have high antibacterial effect when compared to the broad-spectrum TE antibiotic [30,47]. Today, most bacteria are multidrug-resistant [5,48-51]. Natural antioxidants such as ascorbic acid, flavonoids, phenolics, and carotenoids are obtained from *Moringa* leaves [52]. It was reported that they have novel active compounds which possess an antibacterial effect and can overcome the multidrug resistance problem [16,53]. Hence, *M. oleifera* is considered one of the new infection-fighting strategies in controlling multi-drug resistant pathogenic bacteria. As well as, it has a wide safety margin for human and animal consumption.

## Conclusion

In the current study, it can be concluded that the most predominant pyogenic bacteria isolated from camel abscesses were *C. pseudotuberculosis*, *S. aureus*, and *E. coli*. An effective infection control program is needed with a highly effective antibacterial agent/s because antibiotic resistance is common in Egyptian isolates. The bactericidal effect of *M. oleifera* leaf extracts was determined against the isolated bacteria. It further discusses optimal conditions for the extraction of essential compounds responsible for the elimination of pathogenic bacteria.

## Authors’ Contributions

EAF was involved in the disease investigation in the field, planning, sampling, bacterial isolation, and mainly participated in the practical part and writing of the manuscript; ASMA and MMK revised the results, microbiological analysis, and manuscript. All authors wrote, read, and approved the final manuscript.
